# The GenoPred pipeline: a comprehensive and scalable pipeline for polygenic scoring

**DOI:** 10.1093/bioinformatics/btae551

**Published:** 2024-09-18

**Authors:** Oliver Pain, Ammar Al-Chalabi, Cathryn M Lewis

**Affiliations:** Department of Basic and Clinical Neuroscience, Maurice Wohl Clinical Neuroscience Institute, Institute of Psychiatry, Psychology and Neuroscience, King’s College London, London, SE5 9RX, United Kingdom; Department of Basic and Clinical Neuroscience, Maurice Wohl Clinical Neuroscience Institute, Institute of Psychiatry, Psychology and Neuroscience, King’s College London, London, SE5 9RX, United Kingdom; Social, Genetic and Developmental Psychiatry Centre, Institute of Psychiatry, Psychology and Neuroscience, King’s College London, London, SE5 8AF, United Kingdom

## Abstract

**Motivation:**

Polygenic scoring is an approach for estimating an individual’s likelihood of a given outcome. Polygenic scores are typically calculated from genome-wide association study (GWAS) summary statistics and individual-level genotype data for the target sample. Going from genotype to interpretable polygenic scores involves many steps and there are many methods available, limiting the accessibility of polygenic scores for research and clinical application. Additional challenges exist for studies in ancestrally diverse populations. We have implemented the leading polygenic scoring methodologies within an easy-to-use pipeline called GenoPred.

**Results:**

Here, we present the GenoPred pipeline, an easy-to-use, high-performance, reference-standardized, and reproducible workflow for polygenic scoring. It requires minimal inputs and offers various configuration options to cater to a range of use cases. GenoPred implements a comprehensive set of analyses, including genotype and GWAS quality control, target sample ancestry inference, polygenic score file generation using a range of leading methods, and target sample scoring. GenoPred standardizes the polygenic scoring process using reference genetic data, providing interpretable polygenic scores. The pipeline is applicable to GWAS and targets data from any population within the reference, facilitating studies of diverse ancestry. GenoPred is a Snakemake pipeline with associated Conda software environments, ensuring reproducibility. We apply the pipeline to UK Biobank data demonstrating the pipeline’s simplicity, efficiency, and performance. The GenoPred pipeline provides a novel resource for polygenic scoring, integrating a range of complex processes within an easy-to-use framework. GenoPred widens access to the leading polygenic scoring methodology and their application to studies of diverse ancestry.

**Availability and implementation:**

Freely available on the web at https://github.com/opain/GenoPred.

## 1 Introduction

The advent of genome-wide association studies (GWAS) has revolutionized our understanding of the genetic architecture of complex traits and diseases. GWAS identify associations between genetic variants and traits across the genome, providing valuable insights into the biological pathways influencing disease risk and trait variability (Van Rheenen *et al.* 2021, Yengo *et al.* 2022, Adams *et al.* 2024). A key application of GWAS findings is the development of polygenic scores, which aggregate the effects of numerous genetic variants across the genome to estimate an individual’s genetic predisposition to a given trait or disease (Dudbridge 2013, Choi *et al.* 2020). Polygenic scoring has shown promise in predicting disease risk (Khera *et al.* 2018), informing personalized medicine (Fahed *et al.* 2022), and contributing to the understanding of the genetic overlap between phenotypes (Power *et al.* 2015, Pain *et al.* 2018, 2022b).

Despite their potential, the application of polygenic scores in both research and clinical settings faces significant challenges. The process of calculating polygenic scores from genotype data and GWAS summary statistics involves multiple complex steps, including quality control, ancestry inference, generation polygenic scoring files, and target sample scoring (Choi *et al.* 2020, Pain *et al.* 2021). Each of these steps is crucial for the calculation of reliable and interpretable polygenic scores. However, the diversity of methodologies makes it difficult for researchers and clinicians to adopt polygenic scoring widely. This complexity limits the accessibility of polygenic scoring and its potential benefits for understanding and predicting complex traits and diseases.

Recognizing these challenges, we introduce the GenoPred pipeline, a comprehensive, easy-to-use pipeline designed to streamline the polygenic scoring process. The pipeline automates and extends a workflow that we used to compare polygenic scoring methods (Pain *et al.* 2021), integrating leading polygenic scoring methodologies within a reference-standardized framework, facilitating the generation of interpretable and transferable polygenic scores. By simplifying and standardizing the polygenic scoring process, the GenoPred pipeline aims to enhance the accessibility and application of polygenic scores across diverse research and clinical contexts.

This article presents the GenoPred pipeline, detailing its features, implementation, and application to real-world data. We demonstrate GenoPred's performance and utility by applying it to UK Biobank, showcasing its ability to produce reliable polygenic scores, in a computationally efficient manner. As an open-source tool, GenoPred can evolve alongside advancements in polygenic scoring methodologies, ensuring its continued relevance and utility in the field.

In the following sections, we describe the design and functionality of the GenoPred pipeline, demonstrate its performance through application to UK Biobank data, and discuss the implications of our work for the future of polygenic scoring in both research and clinical applications.

## 2 Overview of GenoPred pipeline

The GenoPred pipeline provides a sophisticated workflow designed to facilitate polygenic scoring, leveraging the robustness of Snakemake—a workflow management system—and Conda for software environment management (Mölder *et al.* 2021). This pipeline is engineered to utilize multiple cores for parallel processing and is adaptable for deployment across high-performance computing (HPC) or cloud computing systems, ensuring scalability and flexibility in research applications. We also provide docker and singularity software containers to run the GenoPred pipeline, facilitating the pipeline’s use, particularly within offline environments, where no internet is available when accessing secure data.

### 2.1 Input data processing

GenoPred accepts a range of input formats, minimizing file preparation in advance of running the pipeline. Individual-level genotype data for the target samples, in which polygenic scores are to be calculated, can be provided in PLINK1, PLINK2, BGEN, VCF, or 23andMe format. GWAS summary statistics can have a range of headers, with GenoPred automatically interpreting them. GenoPred automatically detects the genome build of input data, allowing for builds GRCh36 (hg18), GRCh37 (hg19), or GRCh38. If chromosome and base pair information is present, RSIDs are automatically inserted by GenoPred. All inputs are harmonized with the reference genetic data, removing variants that cannot be identified in the reference, updating RSIDs, and resolving strand flips. GenoPred also has the ability to use externally provided score files containing genetic effects to be used for polygenic scoring. The user can specify Polygenic Score Catalog IDs, which GenoPred will automatically download, or the user can provide locally stored score files following the Polygenic Score Catalog header format (Lambert *et al.* 2021).

### 2.2 Ancestry inference

It is important to take into consideration an individual’s ancestry when analysing and interpreting their polygenic scores. GenoPred includes an ancestry inference step, matching target individuals to populations present in the reference dataset. By default, the reference dataset is a combination of samples from 1000 Genomes phase 3 (1KG) (1000 Genomes Project Consortium 2015) and the Human Genome Diversity Project (Bergström *et al.* 2020), capturing a range of global populations, though the user can specify alternative references to be used. The ancestry inference results enable the scaling of polygenic scores according to an ancestry-matched reference, standardizing scores to units of standard deviation (SD) from the mean of the matched reference population.

### 2.3 Leading polygenic scoring methodology

GenoPred integrates seven leading polygenic scoring methods that adjust GWAS effect sizes for polygenic scoring. These include *P*-value thresholding and clumping (pt+clump) (Chang *et al.* 2015), lassosum (Mak *et al.* 2017), DBSLMM (Yang and Zhou 2020), LDpred2 (Privé *et al.* 2020), SBayesR (Lloyd-Jones *et al.* 2019), MegaPRS (Zhang *et al.* 2021), and PRS-CS (Ge *et al.* 2019). The default parameters used for each method are shown in the technical documentation (Supplementary Data File S1). Certain parameters for each method can be altered by the user, such as selecting only the ‘auto’ model within LDpred2. This diversity in methodologies ensures that users can select the approach best suited to their study's needs. The adjusted genetic effects, referred to as score files, are then used to calculate polygenic scores in target samples using PLINK2 (Chang *et al.* 2015).

### 2.4 Principal component estimation

GenoPred enables the calculation of both within-sample or reference-projected principal components (PCs). Within-sample PCs are estimated using the target sample data, which are commonly included as covariates when performing association analyses (Price *et al.* 2006). This process also estimates relatedness within the target sample, which is also important to consider in association analyses. Reference-projected PCs are estimated using the reference dataset, and then projected into the target sample, making them suitable for inclusion in prediction models (Chen *et al.* 2015).

### 2.5 User-friendly output

GenoPred generates detailed reports at both the sample and individual levels (examples in Supplementary Figs S1 and S2). These reports summarize input data processing metrics, ancestry inference results, and the polygenic scores. Individual-level reports provide ancestry inference and polygenic scores for a specific individual, presenting the polygenic scores on both relative and absolute scales to improve interpretability (Pain *et al.* 2022a). The outputs of the pipeline are stored within a simple file structure that is easy to navigate. GenoPred also contains a series of R functions that use the pipeline configuration to read key pipeline outputs directly into R (R Core Team 2015).

### 2.6 Configurable and responsive

Users have the flexibility to trigger comprehensive analyses or request specific outputs of interest, avoiding unnecessary computational steps. For example, the user could request that the pipeline only performs quality control of the GWAS summary statistics, or that the pipeline only applies selected tuning parameters for a given polygenic scoring method. Modifications to input data or configurations prompt the pipeline to automatically update and rerun necessary steps, ensuring that outputs correspond to the latest data and research objectives. Various configuration parameters can be easily set by the user, allowing the user to adjust pipeline behaviour to suit their needs.

**Figure 1.**
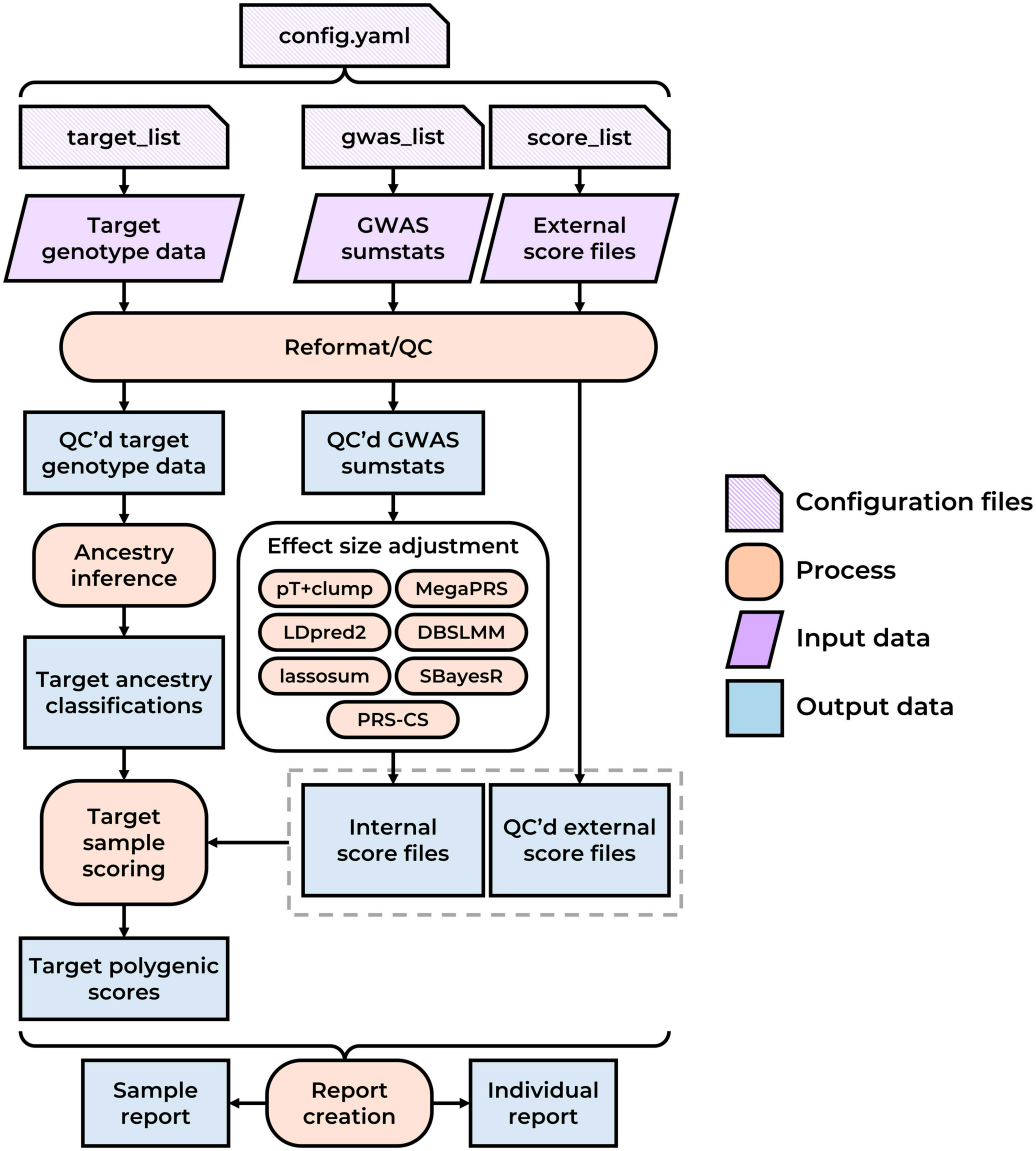
Simplified schematic diagram of the GenoPred pipeline. QC’d = quality controlled.

Figure 1 shows a schematic representation of the pipeline. Technical documentation of each step in the pipeline is provided the Supplementary Data File S1 and online.

## 3. Application to UK Biobank

To demonstrate the simplicity, scalability, and performance of the GenoPred pipeline, we applied it to calculate polygenic scores in the UK Biobank sample (Bycroft *et al.* 2018).

### 3.1 Configuration

**Figure 2.**
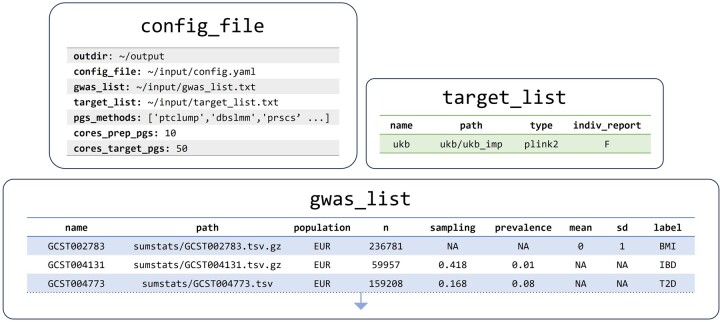
Configuration files instructing GenoPred pipeline to calculate polygenic scores in UK Biobank. Paths are simplified for clarity in this figure. Actual configuration files used are available in Supplementary Tables S2–S4.

We configured the GenoPred pipeline using three files (Fig. 2). These directed GenoPred to compute polygenic scores within the UK Biobank using all 7 polygenic scoring methods across 13 GWAS summary statistics (Supplementary Table S1). The ‘config_file’ specifies the output directory, configuration file locations, and the polygenic scoring methods to be employed. The ‘target_list’ describes the target genetic dataset that polygenic scores should be calculated in. The ‘gwas_list’ describes the GWAS summary statistics that should be used to derive the polygenic scores.

In this study, using GenoPred release v2.2.5, we allowed 10 cores for polygenic scoring methodology and 50 cores for target samples, using the ‘cores_prep_pgs’ and ‘cores_target_pgs’ parameters, respectively.

### 3.2 Execution

Once the configuration files have been prepared, a single line of code can be used to execute the full pipeline:

snakemake --profile slurm --use-conda --configfile=∼/input/config.yaml output_all

The ‘output_all’ command indicates which outputs of the pipeline are to be produced. In this case, it requests the final reports from the pipeline, which triggers all upstream steps of the pipeline (shown in Fig. 1). A range of other commands can be used to produce intermediate or auxiliary outputs (Supplementary Data File S1). The ‘-configfile’ option tells the pipeline which configuration file to use. The ‘-profile slurm’ instructs the pipeline how to distribute the steps using the SLURM job scheduler for our HPC (King’s College London 2022). Snakemake can interact with many different job schedulers or be run locally across cores using the ‘-j’ parameter. The ‘-use-conda’ option tells the pipeline to use Conda environments specified by each step in the pipeline.

### 3.3 Computational resources

The computational resources required vary at different stages of the pipeline and also depend on the pipeline configuration and input data. In this section, we summarize the time required to run each step of the pipeline when using the UK Biobank configuration above. We used the full UK Biobank imputed genotype data in PLINK2 format as input (487 410 individuals, 9.94M variants).

**Figure 3.**
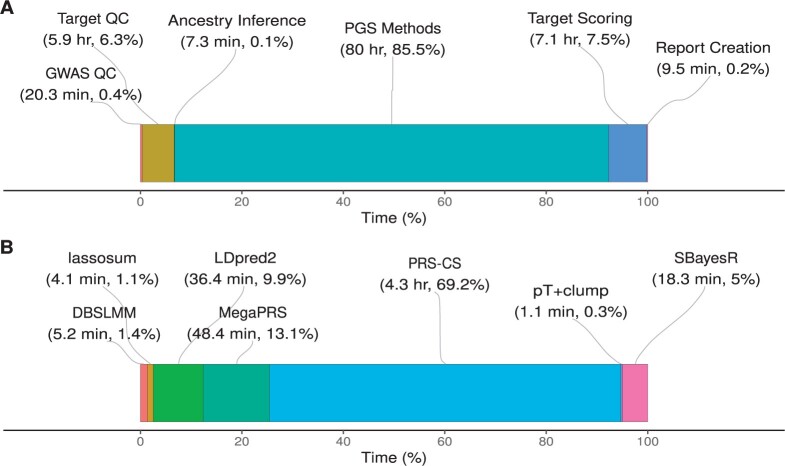
Wall time for steps of the pipeline to complete using the UK Biobank configuration. (A) The total time for each step of pipeline, aggregating the polygenic scoring methods (labelled ‘PGS Methods’). (B) A breakdown of time for each polygenic scoring method, showing the average time per GWAS. QC, quality control; PGS, polygenic scoring.

When using the UK Biobank configuration described above the total computational wall time was 93.57 hours. When an HPC is available, and many steps can be executed in parallel, the time taken for the pipeline to finish is significantly reduced. Most of the time is spent applying polygenic scoring methodology to adjust GWAS effect sizes (85.5%), and then target scoring (7.5%) and target QC (6.3%) (Fig. 3A). The time taken to perform target QC and target scoring is dependent on the number of individuals in the target datasets. The time taken for polygenic scoring methods to run is independent of the target datasets specified, but it will vary depending on which polygenic scoring methods are selected and the number of GWAS.

The time taken by each polygenic scoring method varies substantially (Fig. 3B), and this may be an important factor when deciding the method/s to be used. Using the above configuration, GenoPred allocated 10 cores when running polygenic scoring methods. When using 10 cores, PRS-CS is the slowest of polygenic scoring methods, taking an average of 4.3 hours per GWAS. In contrast, all other methods can be run in <1 hour. The pT+clump, lassosum, and DBSLMM methods are particularly efficient, taking <10 minutes.

### 3.4 Performance

To demonstrate the performance of polygenic scores produced by the GenoPred pipeline, we estimated the association between polygenic scores and 14 relevant phenotypes, using univariate linear and logistic regression for continuous and binary outcomes respectively. Continuous outcomes and polygenic scores were standardized to have a mean of 0 and an SD of 1. The associations were estimated separately for each ancestral population in UK Biobank, as determined by the ancestry inference step of the pipeline (Supplementary Table S5). We used the same GWAS and UK Biobank phenotypes as a recent publication comparing polygenic scoring methodology for comparison (Monti *et al.* 2024), with some minor amendments (Supplementary Information S1). Sample sizes for each phenotype are in Supplementary Table S6.

**Figure 4.**
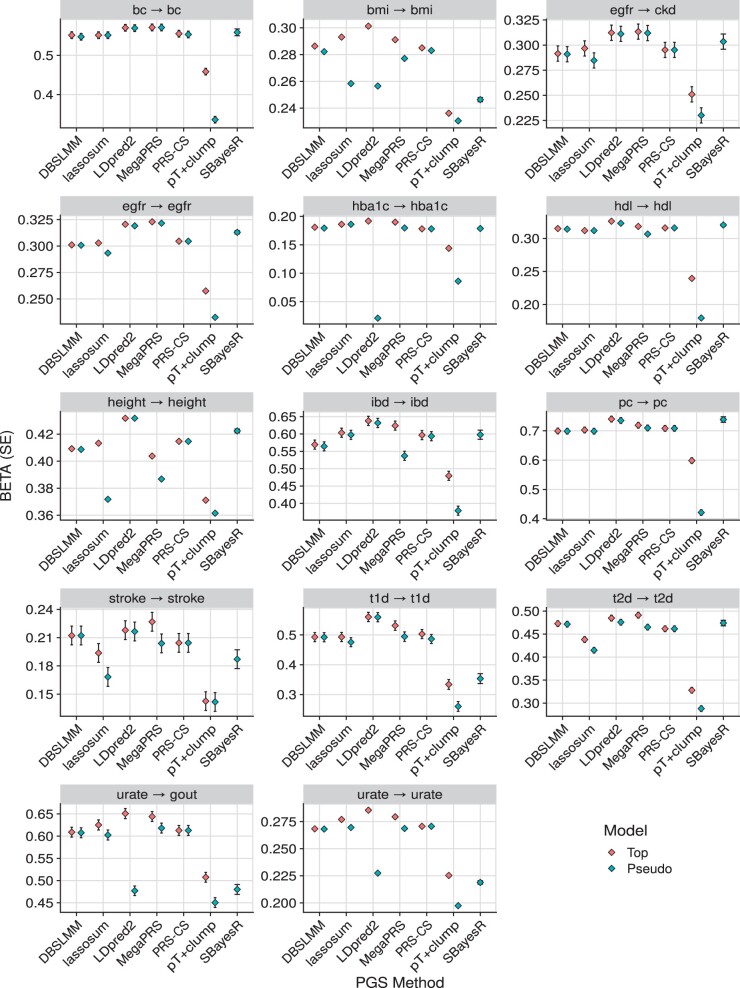
Association between polygenic scores and relevant phenotype data in the European subset of UK Biobank. For binary outcomes, the BETA corresponds to the log(odds ratio). The pseudo model refers to the polygenic score selected by each method’s pseudovalidation method, sometimes referred to as the ‘auto’ model. The pseudo model for the pT+clump method is a *P*-value threshold of 1. The top model is the polygenic score with the largest absolute correlation with the outcome. In the ‘egfr → ckd’ plot, the direction of associations was reversed to ensure the highest values correspond to the best performance in all plots. bc, breast cancer; bmi, body mass index; egfr, estimated glomerular filtration rate; ckd, chronic kidney disease; hba1c, haemoglobin A1c; hdl, high density lipoprotein; ibd, inflammatory bowel disease; pc, prostate cancer; t1d, type 1 diabetes; t2d, type 2 diabetes.

**Figure 5.**
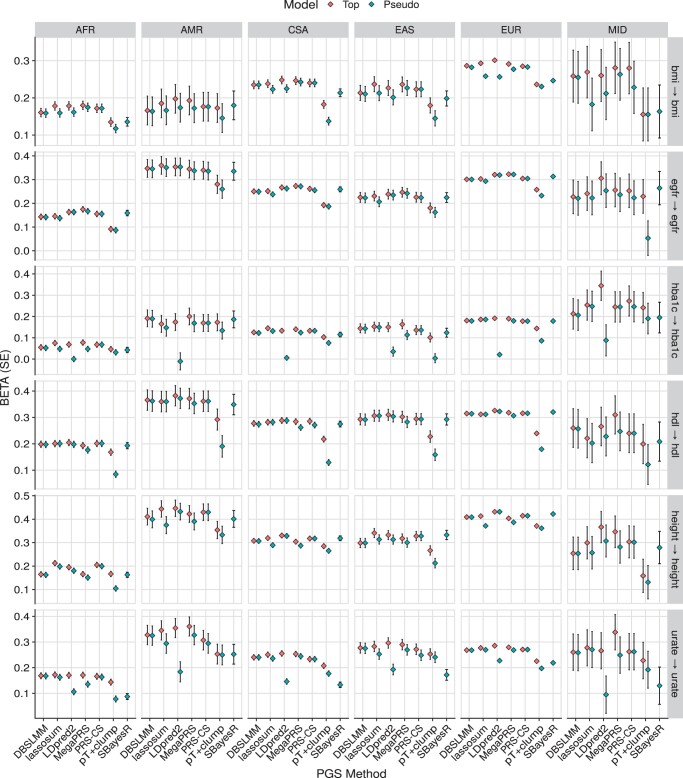
Association between polygenic scores and relevant continuous phenotype data in each UK Biobank population. The pseudo model refers to the polygenic score selected by each method’s pseudovalidation method, sometimes referred to as the ‘auto’ model. The pseudo model for the pT+clump method is a *P*-value threshold of 1. The top model is the polygenic score with the largest absolute correlation with the outcome. AFR, African; AMR, Admixed American; bmi, body mass index; CSA, Central and South Asian; EAS, East Asian; egfr, estimated glomerular filtration rate; EUR, European; hba1c, haemoglobin A1c; hdl, high-density lipoprotein; MID, Middle Eastern.

Figure 4 shows the association results within the European subset of UK Biobank alone, to highlight the relative performance of polygenic scoring methods. Figure 5 shows association results for continuous outcomes in all populations, to highlight relative association across populations. Supplementary Fig. S3 shows results for binary outcomes in all populations. These plots show the association between polygenic scores and the relevant outcome. For example, for body mass index (BMI), a 1 SD increase of the best performing polygenic score from LDpred2, is associated with a 0.3 SD increase in observed BMI. Or for breast cancer, a 1 SD increase of the best performing polygenic score from MegaPRS, is associated with an increase of 0.57 in the log(odds) of breast cancer (corresponding to an odds ratio of 1.77).

Collectively, these results were congruent with the previous study by Monti and colleagues, with regard to the relative performance of each polygenic scoring method, and the absolute performance of the polygenic scores in UK Biobank.

## 4. Discussion

The GenoPred pipeline represents a significant advance in the field of genetic epidemiology, particularly in the application and utility of polygenic scores. By integrating leading methodologies within a user-friendly and reproducible framework, GenoPred facilitates the generation of reliable and interpretable polygenic scores enabling a broader adoption of polygenic scoring in research and clinical settings where specialist computational and statistical expertise may not be available.

Our application of GenoPred to UK Biobank data underscores the pipeline's efficiency, performance, and versatility. The results demonstrate GenoPred’s capability to handle large-scale datasets while maintaining high standards of quality control and ensuring the reproducibility of analyses. Importantly, by standardizing the polygenic scoring process using reference genetic data, GenoPred enhances the interpretability of scores across diverse populations. This is crucial for the application of polygenic scores in global health, as it ensures that the benefits of genetic research are accessible to individuals of varied ancestries.

### 4.1 Comparison to existing tools

To our knowledge, no other publicly available software offers such a comprehensive solution for polygenic scoring as the GenoPred pipeline. While many excellent tools exist for individual steps within the GenoPred pipeline, there is a notable absence of software that seamlessly integrates these steps. We will compare the GenoPred pipeline to several notable tools for polygenic scoring, highlighting the differences.

PRSice-2 is popular for its ease of use and computational efficiency (Choi and O’Reilly 2019). A key feature of PRSice-2 is its ability to perform association analysis and output plots, a function currently not included in the GenoPred pipeline. However, PRSice-2 only includes the pT+clump polygenic scoring method, which underperforms compared to more recent methods (Pain *et al.* 2021). Furthermore, PRSice-2 is less flexible regarding GWAS or target sample data inputs, does not include target sample ancestry inference, and lacks reference-standardization of polygenic scores, making it challenging to analyse and interpret scores in ancestrally diverse target samples.

pgsc_calc is recently developed software primarily for target sample scoring using previously derived score files, stored within the Polygenic Score Catalog or elsewhere (Lambert *et al.* 2024). Like the GenoPred pipeline, pgsc_calc includes an ancestry inference module and standardizes polygenic scores according to an ancestry-matched reference. Additionally, pgsc_calc offers a continuous correction to polygenic scores by regressing out reference-projected PCs, based on their relationship with polygenic scores in a reference dataset (Khera *et al.* 2019). This adjusts for population stratification within reference populations and returns polygenic scores for all individuals, not just those assigned to a reference population. However, pgsc_calc relies on previously derived scoring files, which may not exist for the GWAS of interest, or may not transfer well across samples due to missing genetic variants. In contrast, the GenoPred pipeline has the additional functionality to derive scoring files from GWAS summary statistics using the user’s choice of polygenic scoring method and genetic variants to be considered, substantially broadening its utility.

A range of other excellent tools for handling genetic data and polygenic scoring exist, such as the bigsnpr R package (Privé *et al.* 2018), PLINK2 (Chang *et al.* 2015), and LDAK (Zhang *et al.* 2021). However, these tools focus on specific tasks with a high degree of control and do not offer an end-to-end solution for polygenic scoring.

### 4.2 Limitations

The current version of the GenoPred pipeline (v2.2.5) has two key limitations.

First, GenoPred assigns individuals to reference populations and standardizes target sample scores according to the mean and SD of the ancestry-matched population. While this solution is often sufficient for research contexts where downstream analyses are conducted in a population-specific manner, it leads to the exclusion of admixed individuals and those not well represented by the current reference populations. A potential solution to this limitation is the continuous ancestry adjustment of polygenic scores, as used in some previous studies in diverse populations (Khera *et al.* 2019), and implemented by the pgsc_calc software.

Second, GenoPred does not currently implement polygenic scoring methods that incorporate GWAS for a given outcome from multiple ancestral populations, such as PRS-CSx (Ruan *et al.* 2022) and BridgePRS (Hoggart *et al.* 2024). As genetic data become more widely available from diverse populations, methods incorporating GWAS from multiple populations are outperforming methods that only consider GWAS from a single population. Currently, to incorporate GWAS from multiple populations in GenoPred, users must either meta-analyse the GWAS and specify the majority population, or model polygenic scores from each population separately.

### 4.3 Future directions

As the field of genetic epidemiology continues to evolve, so too will the methodologies for polygenic scoring. GenoPred is well-positioned to adapt to these advances, thanks to its open-source nature and the active community of developers committed to its ongoing development. Future updates will aim to incorporate novel scoring methods, enhance support for multi-ancestry analyses, and improve the integration of functional genomic data to refine the predictive power of polygenic scores.

Moreover, the continued application of GenoPred across diverse research projects will generate valuable insights into its utility and limitations, guiding further refinements. Collaborations with clinical researchers will be particularly important for exploring the potential of polygenic scores to inform clinical decision-making and patient care.

## 5 Conclusion

GenoPred fills a critical gap in the landscape of genetic research tools by providing a standardized, efficient, and accessible pipeline for polygenic scoring. Its development is a step forward in realizing the promise of personalized medicine and underscores the importance of making complex genetic analyses more accessible and interpretable. As we continue to enhance GenoPred and expand its capabilities, we look forward to its contribution to advancing our understanding of the genetic basis of diseases and traits and its application in improving human health.

## Supplementary Material

btae551_Supplementary_Data

## Data Availability

GWAS summary statistics used in this study were publicly available (see Supplementary Table S1). The UK Biobank data was accessed via project 82087—for access, go to https://www.UKBiobankiobank.ac.uk/enable-your-research/apply-for-access. Further information is also provided on the GenoPred pipeline website: https://opain.github.io/GenoPred/pipeline_overview.html.
