# Supplementary material for: The GenoPred pipeline: a comprehensive and scalable pipeline for polygenic scoring
Source: Bioinformatics. 2024 Sep 18;40(10):btae551. doi: 10.1093/bioinformatics/btae551 (PMC11462442; doi:10.1093/bioinformatics/btae551)
Supplement: btae551_Supplementary_Data [file btae551_supplementary_data.zip › GenoPredPipe Bioinf - post_accept -SuppInfo.docx]

# Supplementary Information

## Amendments to Monti et al. GWAS selection and phenotype definitions

We excluded the GWAS for Alzheimer’s disease as its sample overlaps with the UK Biobank target sample in this study. For Height, we used the Yengo et al. GWAS which excludes UK Biobank, allowing us to examine its performance in UK Biobank. The Height phenotype in UK Biobank was defined using the field 50 - Standing height. For RA, we used a more recent GWAS by Ishigaki et al (GCST90132223).

# Supplementary Figure Legends

Figure S1. Example individual-level report produced by the GenoPred pipeline using test data.

Figure S2. Example sample-level report produced by the GenoPred pipeline using test data.

Figure S3. Association between polygenic scores and relevant binary phenotype data in each UK Biobank population (AFR = African, AMR = Admixed American, CSA = Central and South Asian, EAS = East Asian, EUR = European, MID = Middle Eastern). The pseudo model refers to the polygenic score selected by each method’s pseudovalidation method, sometimes referred to as the ‘auto’ model. The pseudo model for the pT+clump method is a p-value threshold of 1. The top model is the polygenic score with the largest absolute correlation with the outcome in each population. In the 'egfr → ckd' plot, the direction of associations was reversed to ensure the highest values correspond to the best performance in all plots. bc = breast cancer, ckd = chronic kidney disease, ibd = inflammatory bowel disease, pc = prostate cancer, t1d = type 1 diabetes, t2d = type 2 diabetes.
