# Supplementary figures and images for: The GenoPred pipeline: a comprehensive and scalable pipeline for polygenic scoring
Source: Bioinformatics. 2024 Sep 18;40(10):btae551. doi: 10.1093/bioinformatics/btae551 (PMC11462442; doi:10.1093/bioinformatics/btae551)

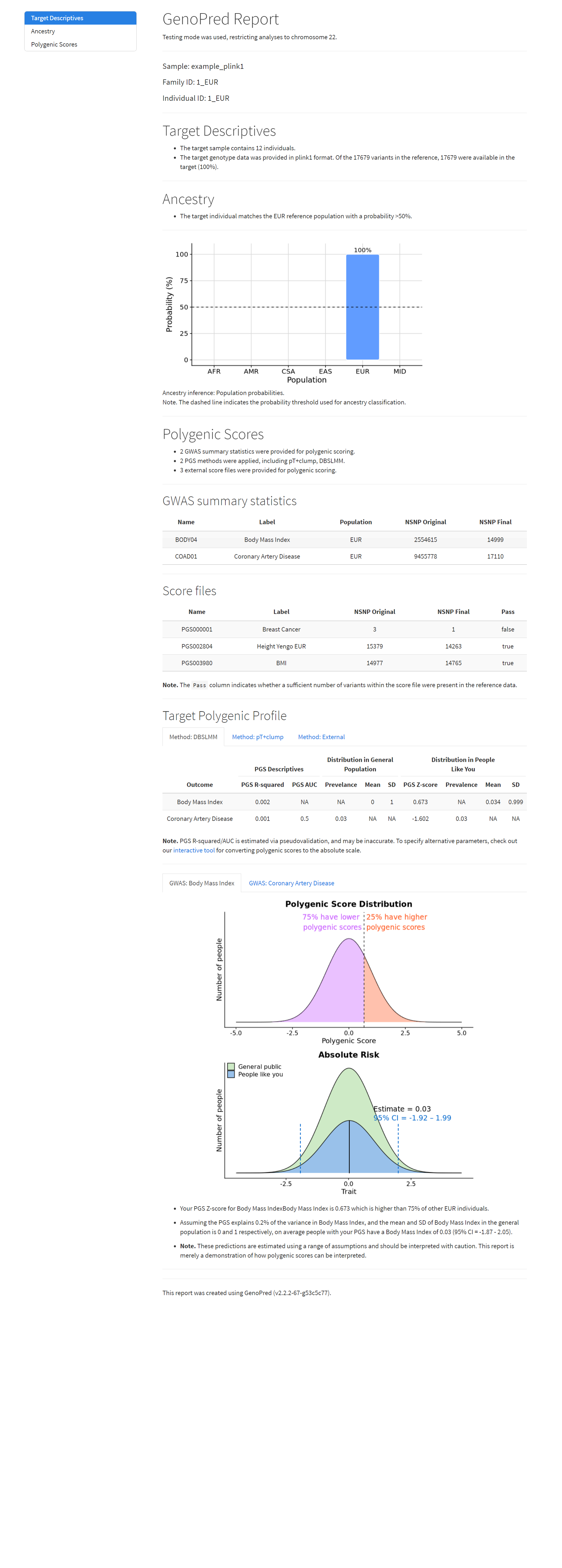

Supplement: btae551_Supplementary_Data [file btae551_supplementary_data.zip › Supplementary Material - Figure S1.png]

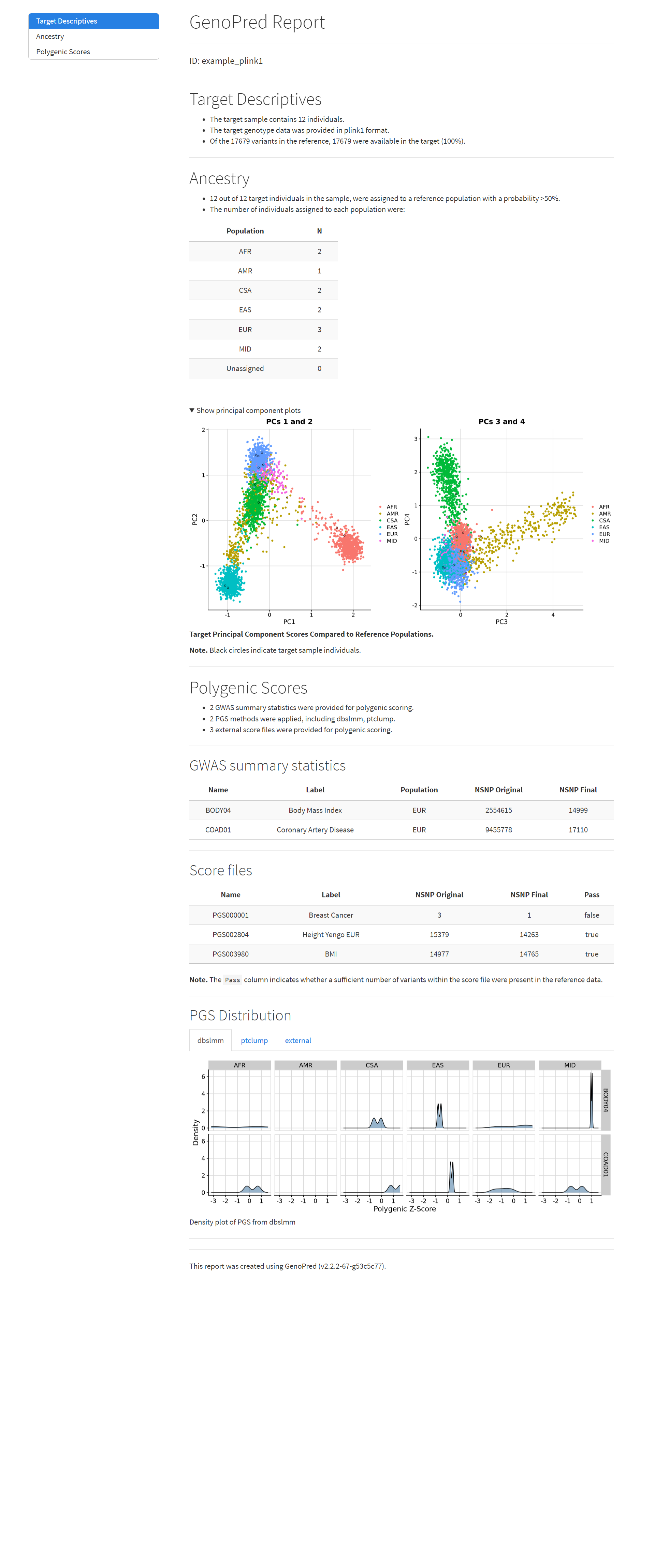

Supplement: btae551_Supplementary_Data [file btae551_supplementary_data.zip › Supplementary Material - Figure S2.png]
